# Differential expression of DHHC9 in microsatellite stable and instable human colorectal cancer subgroups

**DOI:** 10.1038/sj.bjc.6603818

**Published:** 2007-05-22

**Authors:** F Mansilla, K Birkenkamp-Demtroder, M Kruhøffer, F B Sørensen, C L Andersen, P Laiho, L A Aaltonen, H W Verspaget, T F Ørntoft

**Affiliations:** 1 Molecular Diagnostic Laboratory, Department of Clinical Biochemistry, Aarhus University Hospital/Skejby, Brendstrupgaardsvej 100, DK-8200 Århus N, Denmark; 2 Department of Pathology, Aarhus University Hospital, Århus, Denmark; 3 Department of Medical Genetics, University of Helsinki, Biomedicum Helsinki, Haartmaninkatu 8, Finland;; 4 Department of Gastroenterology and Hepatology, Leiden University Medical Center, Leiden, The Netherlands

**Keywords:** DHHC9, immunohistochemistry, microarray, microsatellite instability

## Abstract

Microarray analysis on pooled samples has previously identified *ZDHHC9* (DHHC9) to be upregulated in colon adenocarcinoma compared to normal colon mucosa. Analyses of 168 samples from proximal and distal adenocarcinomas using U133plus2.0 microarrays validated these findings, showing a significant two-fold (log 2) upregulation of DHHC9 transcript (*P*<10^−6^). The upregulation was more striking in microsatellite stable (MSS), than in microsatellite instable (MSI), tumours. Genes known to interact with DHHC9 as H-Ras or N-Ras did not show expression differences between MSS and MSI. Immunohistochemical analysis was performed on 60 colon adenocarcinomas, previously analysed on microarrays, as well as on tissue microarrays with 40 stage I–IV tumours and 46 tumours from different organ sites. DHHC9 protein was strongly expressed in MSS compared to MSI tumours, readily detectable in premalignant lesions, compared to the rare expression seen in normal mucosa. DHHC9 was specific for tumours of the gastrointestinal tract and localised to the Golgi apparatus, *in vitro* and *in vivo*. Overexpression of DHHC9 decreased the proliferation of SW480 and CaCo2 MSS cell lines significantly. In conclusion, DHHC9 is a gastrointestinal-related protein highly expressed in MSS colon tumours. The palmitoyl transferase activity, modifying N-Ras and H-Ras, suggests DHHC9 as a target for anticancer drug design.

Colon cancer is among the most frequent cancers in the Western World with an overall 5 years survival around 50% (Lv *et al*, 2006). Early diagnosis is a key for cancer survival, as early-stage tumours have a better prognosis. Therefore, it is relevant to identify new biomarkers for early lesions. We identified DHHC9 as an early colon cancer biomarker both at the transcript and protein levels.

Two major molecular subgroups of colon cancer exist, microsatellite instable (MSI) and microsatellite stable (MSS) (Fearon and Vogelstein, 1990; Lengauer *et al*, 1997), where MSI tumours represent approximately 15% of the total incidence (Clark *et al*, 2004). Microsatellite instable tumours show mutations or epigenetic alterations in the mismatch repair genes that lead to alterations in microsatellite DNA (short repeated sequences of DNA). Increasing evidence suggests that MSI tumours are associated with better prognosis (Clark *et al*, 2004) and that patients with MSI may not benefit from fluorouracil-based adjuvant chemotherapy (Ribic *et al*, 2003; Benatti *et al*, 2005).

A differential behaviour of single molecules in MSS and MSI tumours may help understanding the molecular basis of these two subgroups and their associated prognostic and therapeutic implications. In the present investigation, we analysed these two subgroups with regard to DHHC9 expression.

In our previous study, microarray expression profiling identified a subset of several hundred expressed sequence tags (ESTs) differentially expressed in pools of distal sporadic colon cancer (Dukes' stages A, B, C and D) when compared to pools of normal mucosa (Birkenkamp-Demtroder *et al*, 2002). One of these was EST AA232508, identified as part of the *ZDHHC9* gene and strongly upregulated in all cancer stages (Birkenkamp-Demtroder *et al*, 2002). Very recently, the protein encoded by the *ZDHHC9* gene, DHHC9, has been characterised. DHHC9 is a 364 amino-acid membrane-bound protein characterised by its Asp-His-His-Cys (DHHC) sequence, within a cysteine-rich domain (Swarthout *et al*, 2005). DHHC9 forms a complex with a Golgi-associated protein of 16 kDa, GCP16. This DHHC9–GCP16 complex has been associated with a protein fatty acyltransferase (PAT) activity, with *in vitro* specificity for H-Ras and N-Ras (Swarthout *et al*, 2005). Palmitoylation of Ras increases the affinity of farnesylated Ras for membranes (Dudler and Gelb, 1996), where Ras can be stably anchored (Berthiaume, 2002). Palmitoylation also facilitates Ras transport (Choy *et al*, 1999; Apolloni *et al*, 2000), which contributes to its biological function in signal-transduction pathways.

In this study, we analysed the expression of DHHC9 mRNA in 168 colorectal adenocarcinomas sampled in Denmark, The Netherlands and Finland, using microarrays. We also studied the protein expression pattern in more than a hundred samples including formalin-fixed paraffin-embedded (FFPE) sections and tissue microarrays (TMAs) using a monospecific polyclonal antibody raised against DHHC9. *In vitro* studies of COS7 cells overexpressing DHHC9 and colocalisation studies on FFPE specimens demonstrated a Golgi-restricted expression of DHHC9.

Overexpression of DHHC9 decreased the proliferation rate of SW480, CaCo2 and DLD-1 with enabled wnt signalling, significantly.

In conclusion, DHHC9 is strongly upregulated in adenocarcinomas of the gastrointestinal tract. The microsatellite status of colon adenocarcinomas distinguishes two highly different molecular subgroups of colon cancer regarding DHHC9 expression.

## MATERIALS AND METHODS

### Clinical specimens

We analysed 168 colorectal samples from patients in Denmark (*n*=108), The Netherlands (*n*=56), and Finland (*n*=14), comprising 122 colon and 46 rectum cancers (Supplementary Table 1). Ten normal mucosa biopsies from the resection edge were collected from Danish patients. Informed consent was obtained from patients, and the local scientific ethical committees approved the study.

### Tissue handling, nucleic acid isolation and microsatellite analysis

The specimens were obtained fresh from surgery and immediately snap-frozen in liquid nitrogen either in an SDS/guanidinium thiocyanate solution or Tissue-Tek OCT compound and subsequently frozen (Denmark), or dry-frozen (Finland and The Netherlands). Total RNA was isolated from the same piece of tissue using Trizol (Invitrogen, Carlsbad, CA, USA). RNA was checked on an Agilent Bioanalyzer and samples with a 28S to 18S ribosomal RNA ratio lower than 1.0 were excluded. The microsatellite status of the tumours was analysed as described previously (Suraweera *et al*, 2002; Kruhoffer *et al*, 2005). Tumours with low-frequency MSI have similar clinical features as MSS tumours and were considered as such in this study.

### Gene expression analysis

Labelling of RNA, hybridisation and scanning were performed as described (Dyrskjot *et al*, 2003). Biotin-labelled cRNA was prepared from 10 *μ*g of total RNA and hybridised to the Human Genome U133plus2.0 GeneChip (Affymetrix, Santa Clara, CA, USA) containing >55 000 probe sets. The readings from the quantitative scanning were analysed by the Affymetrix Software MAS5.0. The resulting cell-files for all 178 samples were imported into ArrayAssist version 3.3 (Stratagene, La Jolla, CA, USA) and data were normalised using GC-RMA (Bolstad *et al*, 2003; Irizarry *et al*, 2003) as implemented in ArrayAssist.

### Statistical analysis

Transcript values were expressed as median log 2±s.d. A two-tailed unequal variance *t*-test was used to determine the differences of DHHC9 expression level in normal colon mucosa *vs* MSS and MSI tumours, and between the MSS and MSI subgroups. Differences with a *P*-value <0.05 were considered as statistically significant.

### Real-time RT–PCR and normalisation

Semiquantitative real-time RT–PCR was performed on 24 samples, four MSI and MSS samples and their corresponding matching normal mucosa as well as four additional MSI and MSS samples with non-matching mucosa. cDNA was synthesised as described (Andersen *et al*, 2004). RT–PCR analysis was performed in triplicates using TaqMan® probe assay ID Hs00211318_m1 (Applied Biosystems) as recommended by the manufacturers and run on a 7500 Fast Real-Time PCR system (Applied Biosystems, Foster City, CA, USA). Results were normalised against UBC, as described previously (Birkenkamp-Demtroder *et al*, 2002; Andersen *et al*, 2004).

### RACE and plasmid construction

RACE was performed using a marathon ready colon adenoma carcinoma cDNA library (Clontech, Mountain View, CA, USA) with primers 5′-UTR-GGGGGACATGTACACTCTTCTTCGC and 3′-UTR-GCCACTGGAGGAAAGTGGAAGTCG.

Wild-type DHHC9 cDNA was PCR amplified from the cDNA library mentioned above using primers sense 5′-GGCAACATGTCTGTGATGGTGGTGAG and antisense 3′-CTACTTCTCAGCTTCAGCTGCCTCC. DHHC9 cDNA was cloned into pcDNA 3.1 bidirectional (Invitrogen) and the DNA sequence was verified. Several normal and colon cancer patients' cDNAs were synthesised from total mRNA using Superscript™ cDNA synthesis kit (Invitrogen).

### Production of monospecific antibodies

Polyclonal rabbit anti-DHHC9 antibodies #EP384 were raised against the peptide CKGSWTGKNRVQNPYS (amino acids 261–274), conjugated to KLH. Antisera were affinity purified against the peptide (Eurogentec, Seraing, Belgium).

### Immunofluorescence microscopy

COS7 cells were cultured on RPMI 1640 medium supplemented with 10% FCS and 1% penicillin–streptomycin at 37°C and 5% CO_2_ and transfected using lipofectamine (Invitrogen), following the manufacturer's instructions. Transfected COS7 cells were fixed and permeabilised with cold methanol (−20°C) at room temperature. Four micrometres FFPE tissue samples were deparaffinised and stained as described previously (Birkenkamp-Demtroder *et al*, 2005). Cells or FFPE specimens were stained with rabbit anti-DHHC9 (1 : 100 cells, 1 : 250 FFPE) and mouse monoclonal anti-58K (1 : 50 cells, 1 : 400 FFPE) (Abcam Ltd, Cambridge, UK). Secondary antibodies were goat anti-rabbit highly cross-adsorbed Alexa®Fluor 488 conjugated (Molecular probes; 1 : 2000 cells, FFPE) in combination with goat anti-mouse Alexa®Fluor 546 conjugated (Molecular probes, Eugene, OR, USA; 1 : 800 cells, 1 : 1600 FFPE). Cells were stained with DAPI for nuclear visualisation. Cells and FFPE tissues were mounted with Fluorescent Mounting Medium DakoCytomation, Glostrup, Denmark. Visualisation was performed using a Zeiss Axiovert fluorescence microscope with 1000 and × 400 magnification or a Leica DMRS confocal microscope with a 64 × HCX PlApo, 1.32 NA objective. Images were merged using Image J and assembled with Adobe Photoshop 9.0.

### Cell proliferation assay

Cell proliferation assays were performed on HCT15, LS174 TR4, HCT116, doxycycline-inducible (1 *μ*g ml^−1^) DLD-1 (van de Wetering *et al*, 2002), CaCo2 and SW480 human colon cancer cells using CyQUANT® NF (Invitrogen). Cell lines were grown as follows: HCT15 in RPMI 1640 medium, SW480 in D-MEM medium, HCT116 in McCoy medium, LS174 TR and DLD-1 in RPMI 1640 medium added HEPES. All cell lines were supplemented with 10% FCS and 1% penicillin/streptomycin. A total of 400–10 000 cells per well were transfected as described above. Dye binding solution (1 × ) was added after 48 h post-transfection and fluorescence intensities were measured using a Biotek FLEX800-TBIDE fluorescence microplate reader at excitation 485/20 nm and detection 528/20 nm. Assays were performed on six replicates per cell line and per plasmid construction.

### Immunohistochemistry

Samples consisted of 60 FFPE biopsies from the superficial non-necrotic part of adenocarcinomas and/or normal mucosa biopsies taken from the macroscopically normal resection margin previously analysed on microarrays (Birkenkamp-Demtroder *et al*, 2002; Kruhoffer *et al*, 2005). Biopsies from hyperplasic polyps, adenomas and normal mucosa from the gastrointestinal tract were taken through endoscopies.

The anti-DHHC9 antibody was applied to 4 *μ*m FFPE sections and stained, as described previously (Birkenkamp-Demtroder *et al*, 2005).

Tissue microarrays were obtained from BioCat GmbH (Heidelberg, Germany). A ‘Colon Cancer Tissue Array COCA912-5-OL (UICC staging)’ and a TMA with human tumours from multiple organ sites and normal specimens from the same sites (T8235713-5-BC) were stained for DHHC9 protein expression using a 1 : 250 dilution of the rabbit anti-DHHC9 antibody. Scoring of the TMAs was independently performed by two experienced investigators (FBS and KBD).

### Cell extraction, SDS gels and Western blots

Transfected COS7 cells were harvested and lysed in lysis buffer (50 mM Tris-HCl pH 8.0, 150 mM NaCl, 1 mM DTT, 1% Triton X-100 and protease inhibitor ROCHE complete, EDTA free). Total protein samples (10–20 *μ*g) were run on 12% SDS gels (Invitrogen) and transferred to nitrocellulose membranes. Membranes were blocked with 3% w/v non-fat powder milk PBS. The primary antibody was rabbit polyclonal anti-DHHC9 (1 : 100) and the secondary antibody goat anti-rabbit HRP conjugated (1 : 5000) (DakoCytomation). The immunoreactive bands were visualised using ECL plus (Amersham biosciences, Piscataway, NJ, USA) and a UVP ChemiDoc-It, Imaging system (UVP Inc., Upland, CA, USA).

## RESULTS

### Microarray and RT–PCR analysis

**Figure 1:**
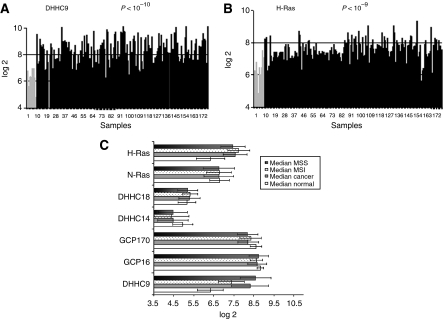
Microarray analysis of 168 samples representing molecular subgroups of CRCs. Transcript expression level of (**A**) DHHC9 and (**B**) H-ras on the U133plus2.0 Gene Chip. The black bars correspond to CRC samples, the grey bars to normal mucosa. Expression values are given as log 2 values and all data are normalised. (**C**) MSS/MSI study. Median log 2 values and standard deviations of normal biopsies (median normal, *n*=10), CRC patients (median cancer, *n*=168), MSI (CRC patients with MSI, *n*=35), MSS (CRC patients with MSS, *n*=118).

Genome-wide expression profiling monitored the presence of several transcripts corresponding to DHHC9 and proteins described to interact with it, or sharing an evolutionary relationship (Fukata *et al*, 2004). We found the DHHC9 transcript to be overexpressed in colorectal cancer (CRC). We found a significant two-fold log 2 increase (*P*<10^−6^) in transcript levels, when adenocarcinoma tissue samples were compared to normal mucosa samples (Figure 1A and Table 1). GCP16, GCP170 (Ohta *et al*, 2003), DHHC14 (Fukata *et al*, 2004), DHHC18 (Fukata *et al*, 2004) and N-Ras, genes known to be related to DHHC9, showed a moderate decrease or no change (Table 1 and Supplementary Figure 1). H-Ras transcription was upregulated in CRC (1.3-fold change, *P*<10^−4^) (Figure 1B and Table 1), confirming previously published data (Feng *et al*, 2001).

Out of 168 patient samples used in our microarray profiling study, 118 were identified as MSS, and 35 were MSI. The remaining 15 samples were of unknown status.

Data analysis pointed out that DHHC9 transcript overexpression differed significantly between MSS and MSI tumours (*P*<10^−13^), as depicted in Figure 1C. The MSS tumours (median log 2, 8.6) showed a 2.3-fold log 2 increase (*P*<10^−7^) of DHHC9 transcript when compared to normal mucosa, while MSI tumours (median log 2, 7.3) showed a 1.0-fold log 2 increase (*P*<10^−4^). In contrast to the DHHC9 transcript, the H-Ras transcript showed a 0.3-fold log 2 increase between MSI and MSS samples (Figure 1C).

Real-time RT–PCR was consistent with those data as *ZDHHC9* transcript expression was only slightly increased in MSI tumours (median 1.1) but highly increased in MSS tumours (median 1.7) when compared to matching normal mucosa samples (median 0.4), as shown in Supplementary Figure 2.

### DHHC9 protein expression pattern in colon adenocarcinomas, distant metastases and TMAs

**Figure 2:**
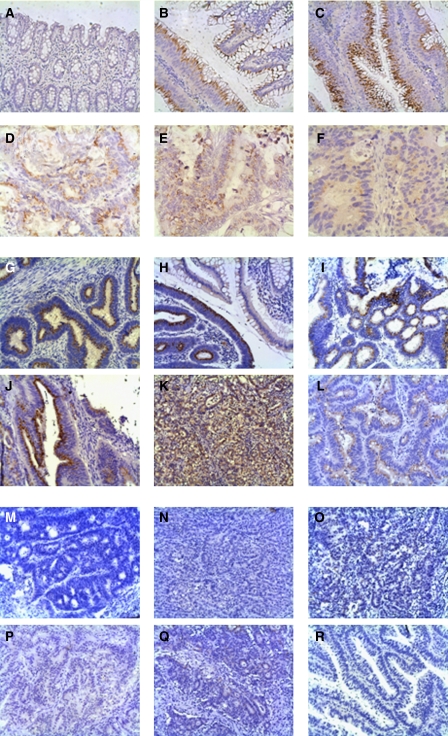
Immunohistochemical analysis of DHHC9 expression. (**A**) normal mucosa, (**B**) Dukes A adenocarcinoma adjacent to premalignant tissue, (**C**) Dukes A adenocarcinoma ( × 200), (**D**) Dukes D adenocarcinoma, (**E**) lymph node metastasis and (**F**) liver metastasis ( × 400). DHHC9 protein expression in MSS *vs* MSI colon adenocarcinomas. Microsatellite stable tumours (**G**–**L**), MSI-H tumours (**M**–**R**) ( × 200).

Immunohistochemical analyses were performed on FFPE adenocarcinomas and their matching normal mucosa, all samples were previously expression profiled (Birkenkamp-Demtroder *et al*, 2005; Kruhoffer *et al*, 2005). Matching normal mucosa was either scored negative for DHHC9, or showed a very weak DHHC9 expression in the cytoplasm of the luminal part of the mucosa as shown in Figure 2A and in more detail in Supplementary Figure 3.

DHHC9 protein was strongly upregulated in adenocarcinomas, thereby accumulating supranuclear, probably in the Golgi apparatus (Figure 2B and C)

To investigate whether DHHC9 was differentially expressed in MSS compared to MSI tumours, immunohistochemical analyses were performed on a subset of 34 colon adenocarcinomas with known MSS/MSI status, previously expression profiled on U133A2.0 arrays (Kruhoffer *et al*, 2005). The subset comprised 16 MSS and 18 MSI tumours and their matching normal mucosa. Eighty-one per cent of the MSS tumours were positive for DHHC9, showing a supranuclear accumulation of DHHC9 (Figure 2G–L), while 77% of the MSI tumours were negative for DHHC9 (Figure 2M–R). Supplementary Figure 3 shows the staining results of additional MSI (Supplementary Figure 3A–G) or MSS (Supplementary Figure 3H–M) tumours, and their matching normal mucosa. The scoring results are summarised in Table 2, and in more detail in Supplementary Table 2.

Remarkably, upregulation of the DHHC9 protein was also detected in early lesions such as benign hyperplasic polyps and tubular and tubulo-villous adenomas (data not shown), as well as at premalignant sites in the colon mucosa (Figure 2B and Supplementary Figure 3N and O) These sites were either located adjacent to, or in close proximity to, adenocarcinomas, for example, detected at the transformation site (Supplementary Figure 3Q). Moreover, accumulation of DHHC9 protein was also identified in three lymph node metastases and 24 liver metastases analysed. An example showing an adenocarcinoma and two metastases from the same patient can be seen in Figure 2D–F.

To investigate whether differential expression of DHHC9 is stage dependent, we analysed a colon cancer TMA comprising adenocarcinomas of the UICC stages I–IV (Table 3). All 10 adenomas and 70–80% of the four UICC stages of adenocarcinomas and liver metastases stained positive for DHHC9. Eighty per cent of the normal mucosa samples were negative, only one was moderately expressing the DHHC9 molecule, as shown in Supplementary Figure 4.

In conclusion, DHHC9 is significantly upregulated in the majority of MSS tumours at both the transcript and protein level. Upregulation is detected at premalignant sites and in very early lesions and is independent of stage and grade.

### DHHC9 expression is confined to tumours of the gastrointestinal tract

We analysed the DHHC9 protein expression pattern in 94 samples comprising 46 cancers from different organ sites and 46 normal tissues using a multiple cancer TMA. DHHC9 was either moderately or not expressed in normal tissues of the body, as shown in Supplementary Figure 5. Results are summarised in Supplementary Table 3. Most common cancers such as, bladder, prostate, breast, kidney, uterus, thyroid, lymph node, tongue or brain were completely negative for DHHC9 expression (Supplementary Figure 5). In contrast, all gastrointestinal adenocarcinomas from the colon rectum, small intestine and stomach showed a very strong positive Golgi-like staining for DHHC9 (Supplementary Figure 5B5, F7, F11 and G7). Moreover, analyses of additional 10 normal mucosa specimens derived from the gastrointestinal tract of healthy people (oesophagus, stomach, small intestine, appendix, colon and rectum) were also negative for DHHC9 expression (data not shown).

### Subcellular localisation of DHHC9 in colon adenocarcinomas and transient overexpression of wild-type DHHC9

**Figure 3:**
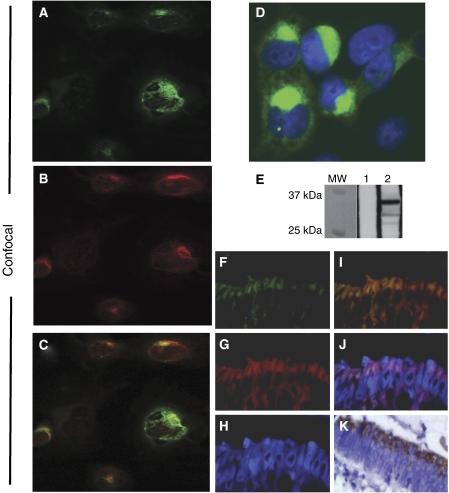
Immunostaining and Western blotting of transiently transfected COS7 cells and immunofluorescence applied to an MSS adenocarcinoma. (**A–C**) Confocal microscope, (**D**) Zeiss Microscope: × 100. (**A**) DHHC9, (**B**) endogenous 58K protein, (**C**) merged DHHC9 and 58K protein, (**D**) DHHC9 Golgi localisation detail. DAPI-stained nuclei are blue. (**E**) Western blotting of extracts from DHHC9-transfected COS7 cells (MW, molecular weight marker; 1, cells transfected with an empty vector; 2, cells transfected with wild-type DHHC9). (**F–K**) IF, (**F**) DHHC9, (**G**) 58K protein, (**H**) DAPI, (**I**) merge (**F**) and (**G**); (**J**) merge (**F**), (**G**) and (**H**); (**K**) DAB staining of the same tumour.

Colocalisation studies using immunofluorescence microscopy were applied to FFPE tissue samples of adenocarcinomas with MSS status. DHHC9 colocalised with the Golgi marker 58K in the Golgi apparatus of the tumour cells, as shown in Figure 3F–K.

The coding cDNA derived from Affymetrix probe set 222451_s_at (DHHC9) was amplified from a commercial colon adenocarcinoma cDNA library or normal and colon cancer total cDNAs. The sequences showed no mutations (data not shown). A 5′- and 3′-UTR RACE amplified DHHC9 transcript variant 1 (NM_016032), proving the expression of a full-length transcript in CRC tissue.

Wild-type DHHC9 transiently overexpressed in COS7 cells colocalised with the Golgi marker 58K to the Golgi apparatus (Figure 3A and D). This coincides with recent findings of Golgi-localised DHHC9 in the embryonic kidney cell line HEK293 (Swarthout *et al*, 2005) and the localisation in colon adenocarcinomas described above.

The specificity of the rabbit anti-DHHC9 antibody was confirmed by Western blot analysis, identifying a 36 kDa band of recombinant wild-type DHHC9 (Figure 3E).

Interestingly, the apparent molecular weight was 5 kDa lower than that of the calculated molecular weight of 40.9 kDa. This suggested that a cleavage of approximately 43–50 amino acids may have taken place affecting the very basic N-terminus of the protein, close to the predicted anchor signal (Lys35-Glu56). No bands were detected in the mock protein cell extract from COS7 cells transfected with an empty vector. There were no signs of cell morphological changes upon DHHC9 transfection.

### DHHC9 expression impacts proliferation of colon cancer cell lines

**Figure 4:**
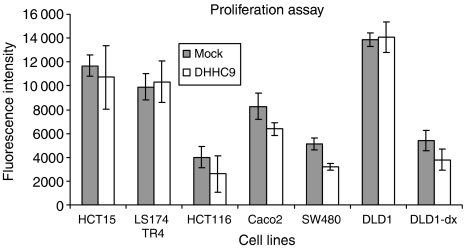
Proliferation assay. Several different cell lines with MSS (SW480, CaCo2) or MSI (HCT15, HCT116, LS174 TR4, DLD-1) were transfected with DHHC9 or a mock empty vector. Forty-eight hours post-transfection cells were added to 1 × dye binding solution, and fluorescence was measured. Only MSS cell lines SW480 and CaCo2 showed a significantly decreased proliferation rate. Interestingly, also DLD-1 cells, with the wnt pathway switched off, showed a similar decreased proliferation rate.

Six colon cancer cell lines with MSS or MSI status were transiently transfected with DHHC9 or a mock empty vector. Proliferation was found to be decreased in SW480 and CaCo2 cell lines (MSS), while HCT15, LS174 TR4 and HCT116 (MSI) were not affected, as shown in Figure 4. All cell lines have an activated wnt-signalling pathway. To investigate the effect of DHHC9 on cell proliferation in a cellular model, we used modified DLD-1 cells where the beta-catenin/TCF4 activity can be disrupted by overexpression of dominant-negative TCF induced by doxycycline (van de Wetering *et al*, 2002). DHHC9 overexpression did not impact proliferation of DLD-1 cells with active wnt signalling. Remarkably, in addition to the previously described effects in SW480 and CaCo2, DHHC9 decreased the proliferation in cells with enabled wnt signalling compared to mock-treated cells.

## DISCUSSION

Microarray analysis on 178 samples showed a significant upregulation of the DHHC9 transcript in individual colorectal tumour samples, when compared to normal mucosa. The upregulation of the DHHC9 transcript as well as the DHHC9 protein was mainly seen in MSS tumours.

The only published data on DHHC9 showed abundant transcript in the kidney, skeletal muscle, brain, lung and liver and moderate expression in the thymus, spleen and peripheral blood leucocytes based on a Northern blotting (Swarthout *et al*, 2005). In contrast, SAGE anatomic viewer at NCBI, based on small 10 bp tags, showed a very low expression of the DHHC9 transcript in almost all the tissues tested (http://cgap.nci.nih.gov/SAGE). Our TMA-based data showed that, apparently, only tumours of the gastrointestinal tract were able to express the protein stably, and most of the other tissues seemed to be consistent with the transcriptional SAGE anatomic viewer profile.

Sporadic CRCs develop as a result of defects in pathways involving either chromosomal instability (Fearon and Vogelstein, 1990) or microsatellite instability (Lengauer *et al*, 1997).

Although the DHHC9 transcript was identified to be slightly upregulated in MSI tumours compared to normal mucosa, our results showed that DHHC9 transcript levels were significantly higher in MSS compared to MSI tumours.

DHHC9 has no repeats in the coding or promoter 5′-UTR regions. Sequencing of seven samples did not detect mutations in any of the amplified DHHC9 cDNAs from several patients (data not shown), leading to the conclusion that the coding sequence was not affected by mismatch repair failures.

It is well known that MSS tumours are genomic instable compared to MSS tumours.

Recently, genome-wide differences between MSS and MSI colorectal tumours have identified the loss of Xq in 31% of the MSS cases studied (Camps *et al*, 2006). We therefore investigated the *ZDHHC9* Xq26.1 locus. Our own analyses of 15 laser microdissected colon adenocarinomas using Affymetrix Mapping 10K SNP arrays (Andersen *et al*, 2007) did not show loss or gain of chromosomal material at Xq26.1. There was no significant difference between male and female patients (*P*=0.15). We conclude that the cause of DHHC9 upregulation and the difference in DHHC9 transcript level between MSS and MSI specimens is not directly related to a chromosomal imbalance at the Xq26.1 locus.

We have previously reported a differential gene expression in proximal compared to distal CRC tumours (Birkenkamp-Demtroder *et al*, 2005). In the present study, there was no significant expression difference between the proximal and distal colon with regard to the DHHC9 transcript. However, there was a tendency for MSI tumour samples to be originated in the right side, while MSS tumours are mostly left sided, as previously reported (Sugai *et al*, 2006).

DHHC9 transcripts were slightly upregulated in MSI tumours compared to normal mucosa. However, the protein itself was weakly expressed or absent in 77% of the MSI tumour samples. We may therefore consider a very rapid turnover of the protein, so it is not detectable, or there may be a tight regulation of the transcript, and consequently no translation will occur in MSI tumours.

Interestingly, proliferation of MSS cell lines SW480 and CaCo2, 48 h post-DHHC9 transfection, showed a slight but significant decrease of the proliferation rate. In contrast, DHHC9 overexpression had no impact on the proliferation of MSI cell lines HCT116, HCT15, LS174 TR4 and DLD-1. All these cell lines have a constitutive active wnt-signalling pathway.

As expected, abrogation of wnt signalling significantly reduced proliferation in DLD-1 cells (van de Wetering *et al*, 2002). However, it cannot be excluded that part of the proliferation decreasing effect can be attributed to doxycycline treatment alone, as previously reported (Onoda *et al*, 2006). Surprisingly, the combination of wnt abrogation with a simultaneous DHHC9 overexpression yielded an even more dramatic reduction in proliferation than the wnt abrogation alone. These results may suggest a potential involvement for DHHC9 in the control of cellular proliferation. As a hypothesis, DHHC9 upregulation after a cellular damage may contribute to an arrest in the cell cycle. This arresting effect seemed to be associated to the MSS status, as only SW480 and CaCo2 transfected cells showed a decreased proliferation rate. Later on, other factors may probably counteract the antitumourigenic effect of DHHC9. Our data suggested that the potential effect of DHHC9 overexpression was independent of an active wnt-signalling pathway and had a synergistic effect upon abrogated wnt-signalling conditions. In addition, the highly tumourigenic, poorly differentiated SW480 cell line and the well-differentiated CaCo2 showed a comparable decrease of the proliferation rate upon DHHC9 overexpression.

The function and location of the protein may suggest the implications of the DHHC9 expression in tumours. We localised the DHHC9 protein in the Golgi apparatus in both colon adenocarcinomas and transfected COS7 cells.

The DHHC9 protein has recently been described as a protein acyl transferase PAT specific for N-Ras and H-Ras (Swarthout *et al*, 2005). *In vitro,* DHHC9 and GCP16 are needed to carry out the palmitoyl transferase activity. Our transcript profiling identified DHHC9 transcript levels to be highly upregulated in MSS tumours compared to normal mucosa while GCP16 and N-Ras showed no differential expression in CRC. It is tempting to suggest that high DHHC9 levels of expression may have an impact on N-Ras or H-Ras affecting its trafficking, modifying their acylation rate cycle regulation (Rocks *et al*, 2005) or affecting clusters of GTP bound, palmitoylated H-Ras and N-Ras, the so-called rasosomes (Rotblat *et al*, 2006).

In this context, proteins that are responsible for post-translational modifications are particularly suitable as potential anticancer drug targets (Leonard, 1997; Waddick and Uckun, 1998). Palmitoylation of N-Ras and H-Ras, a post-translational modification that takes place right after farnesylation, could become a new target. Unfortunately, although PAT activity is well known, the enzymes responsible for it are still elusive. However, the fact that DHHC9 has been postulated to be a PAT specific for both N-Ras and H-Ras, together with our results showing specific overexpression of the protein in gastrointestinal cancers already at the early stages of the disease, could be a first opening.

The role of DHHC proteins in cancer is just emerging. In this study, we bring forth a new perspective on *ZDHHC9*. We showed that *ZDHHC9* is significantly increased in CRC at the transcript and protein levels reflecting a significant difference between MSS and MSI tumours. In addition, the postulated specific PAT function of DHHC9, modifying N-Ras and H-Ras, suggests a very interesting potential as anticancer drug target.

## Figures and Tables

**Table 1:** Summary of microarray transcript profiling

	**Median log 2**				**MSS/MSI**
	**N**	**T**	**Log 2 fold change**	***P*-value**	***P*-value**
DHHC9	6.3	8.3	2.0	<10^−6^	<10^−13^
GCP16	8.8	8.7	−0.1	0.06	0.07
GCP170	8.6	8.2	−0.4	<10^−4^	0.003
N-Ras	6.7	6.7	0	0.964	0.61
H-Ras	6.3	7.6	1.3	<10^−4^	0.008
DHHC14	5.0	4.5	−0.5	0.01	0.61
DHHC18	5.2	5.3	−0.1	0.18	0.002

MSI=microsatellite instable; MSS=microsatellite stable.

Data are given as median log 2, fold change between normal (N) and tumour (T) and *P*-value.

**Table 2:** Immunohistochemical analysis of DHHC9 protein expression applying a 1 : 250 dilution of anti-DHCC9 antibody on 34 CRC tissue samples

	**Negative**	**Weak**	**Total**	**Moderate**	**Strong**	**Very strong**	**Total**
MSS	3	0	3	3	5	5	13
MSI	8	6	14	1	1	2	4
							
	**Negative or weak expression**				**Moderate to strong expression**		
MSS	3/16		18.75%		13/16		81.25%
MSI	14/18		77.78%		4/18		22.22%

CRC=colorectal cancer; MSI=microsatellite instable; MSS=microsatellite stable.

The intensity is classified as negative (comprising negative or weak) and positive (comprising moderate, strong or very strong).

**Table 3:** Immunohistochemical analysis of DHHC9 protein expression applying a 1 : 250 dilution of anti-DHCC9 antibody to a colon cancer TMA

	**Normal mucosa**	**Adenoma**	**ADC I**	**ADC II**	**ADC III**	**ADC IV**	**Liver metastases**
Total cases	10	10	10	10	10	10	11
Average age (years)	69	64	69	75	72	62	64
							
Male	6	5	5	2	6	4	7
Female	4	5	5	8	4	6	4
							
*Staining*							
Negative	8	0	2	2	2	3	3
Positive	2	10	8	8	8	7	8
Strong	1	3	5	1	3	2	4
Moderate	1	7	3	7	5	5	4

TMA=tissue microarray.

The intensity of the positive cases is further classified as moderate or strong.
