# Supplementary material for: Differential expression of DHHC9 in microsatellite stable and instable human colorectal cancer subgroups
Source: Br J Cancer. 2007 May 22;96(12):1896–903. doi: 10.1038/sj.bjc.6603818 (PMC2359975; doi:10.1038/sj.bjc.6603818)
Supplement: Supplementary Figure Legend [file 6603818x6.doc]

**Figure legends to Supplementary figures**

Supplementary Figure 1:

Microarray analysis of 168 patient samples representing common groups of colorectal cancers. Transcript expression levels of five genes selected from the more than 55000 probe sets on the U133plus2.0 arrays are represented here. The black bars correspond to colorectal cancer patients’ values, the grey bars to normal mucosa biopsies. Log2 values are normalised and the protein names are chosen in each graph for ease of interpretation. a) GCP170, b) GCP16, c) N-Ras, d) DHHC18, e) DHHC14.

Supplementary Figure 2

Real-Time PCR analysis of RNA from CRC tumours. Each column represents a single sample. Four MSI (Black) and MSS (white) tumour samples named T1-T4, T9-T12 are represented together with their normal matching mucosa (grey) named N1-N12. In addition, four unmatched MSI and MSS samples, T5-T8 and T13-T16, are also shown. The amount of DHHC9 transcript has been normalised against ubiquitin.

Supplementary Figure 3

Immunohistochemical analysis of FFPE sections of tumours with MSS and MSI status (20x magnification)

a)-m) Normal colon mucosa (Normal, left) from the resection edge and matching adenocarcinoma (Tumour, right) from the same patient. Arrows indicate sites with DHHC9 expression.

a)-g) MSI tumours showing low or no expression of DHCC9

h)-m) MSS tumours showing strong expression of DHCC9

n) and o) Pre-malignant tissue samples with upregulation of DHHC9

p) MSI tumour with focal high DHHC9 expression

q) Transformation site showing an MSS tumour and adjacent pre-malignant tissue with high DHHC9 expression

r)-u) Samples from a single patient, containing two adjacent tumours. Microsatellite analysis performed on a biopsy from tumour one (T1) defined an MSI tumour. Immunohistochemical analysis identified two tumours adjacent to each other with different morphology and different DHHC9 expression.

r) Adenocarcinoma with very low DHHC9 expression (20x), morphology similar to MSI tumours.

s) Normal mucosa (N) with adjacent tumour one (T1) (5x)

t) Normal mucosa (N) with adjacent tumour two (T2) (5x)

u) Adenocarcinoma with very high DHHC9 expression(20x), morphology similar to expression in MSS tumours.

Supplementary Figure 4

Immunohistochemical analysis of DHHC9 expression on a colon tissue microarray containing 40 colon adenocarcinomas with UICC staging I-IV, 10 adenomas, 10 normal mucosa and 11 liver metastases (only 10 shown here).

Supplementary Figure 5

Immunohistochemical analysis of DHHC9 expression applying a 1:250 dilution of anti-DHCC9 antibody to a multiple cancer TMA. A subset of 32 samples is described below.

B5 colon tumour (adenocarcinoma), F7) rectum tumour (adenocarcinoma), F11) small intestine tumour (adenocarcinoma) and G7) stomach tumour (adenocarcinoma) were staining strongly positive for DHHC9 while all other arrayed cancers were negative for DHHC9: A5) adipose tumour (liposarcoma), A6) normal adipose tissue; A7) bladder tumour (transitional cell carcinoma), A8) normal urothelium; A11) brain tumour (astrocytoma), A12) normal brain; B1) breast tumour (invasive ductal carcinoma), B2) normal breast; C3) oesophagus tumour (adenocarcinoma), C4) normal oesophagus; C11) kidney tumour (granular cell carcinoma), C12) normal kidney; D9) lung tumour (adenocarcinoma), D10) normal lung; E3) lymphoma, E4) normal lymph node; E7) ovary tumour (adenocarcinoma), E8) normal ovary ; F3) prostate tumour (adenocarcinoma), F4) normal prostate; G11) tongue tumour (Squamous Cell Carcinoma), G12) normal tongue; H11) uterus tumour (adenocarcinoma), H12) normal uterus; H5) thyroid tumour (adenocarcinoma), H6) normal thyroid.

B8) normal colon mucosa stained weakly positive at the luminal part of the mucosa.
