# Supplementary material for: Differential expression of DHHC9 in microsatellite stable and instable human colorectal cancer subgroups
Source: Br J Cancer. 2007 May 22;96(12):1896–903. doi: 10.1038/sj.bjc.6603818 (PMC2359975; doi:10.1038/sj.bjc.6603818)
Supplement: Supplementary Table 1 [file 6603818x7.doc]

**Supplementary Table 1**:

Microarray analysis resume table. a) Clinical specimens gender, age, Dukes’ stages, grade and MSS/MSI status.

| Tumour  specimens |  | 168 |  | Normal specimens |  | 10 |
| --- | --- | --- | --- | --- | --- | --- |
|  |  |  |  |  |  |  |
| Gender | Male | 91 |  | Gender | Male | 8 |
|  | Female | 77 |  |  | Female | 2 |
|  |  |  |  |  |  |  |
| Age | < 50 years | 12 |  | Age |  |  |
|  | 50-70 years | 89 |  |  | 50-70 | 6 |
|  | > 70 years | 67 |  |  | >70 | 4 |
|  |  |  |  |  |  |  |
| Dukes’ Stages | A | 1 |  |  |  |  |
|  | B | 149 |  |  |  |  |
|  | C | 13 |  |  |  |  |
|  | D | 5 |  |  |  |  |
|  |  |  |  |  |  |  |
| Grade | 1 | 20 |  |  |  |  |
|  | 2 | 115 |  |  |  |  |
|  | 3 | 30 |  |  |  |  |
|  | N.D. | 3 |  |  |  |  |
|  |  |  |  |  |  |  |
| MSS/MSI | MSS | 118 |  |  |  |  |
|  | MSI | 35 |  |  |  |  |
|  | N.D. | 15 |  |  |  |  |
