# Supplementary material for: Differential expression of DHHC9 in microsatellite stable and instable human colorectal cancer subgroups
Source: Br J Cancer. 2007 May 22;96(12):1896–903. doi: 10.1038/sj.bjc.6603818 (PMC2359975; doi:10.1038/sj.bjc.6603818)
Supplement: Supplementary Table 2 [file 6603818x8.doc]

**Supplementary Table 2:** Immunohistochemical analysis of 16 MSS and 18 MSI-H colon adenocarcinomas applying a 1:250 dilution of the polyclonal anti-human DHHC9 antibody. Matching normal mucosa from the resection edge was analyzed for 14 samples.
